# Submergence Gene *Sub1A* Transfer into Drought-Tolerant *japonica* Rice DT3 Using Marker-Assisted Selection

**DOI:** 10.3390/ijms222413365

**Published:** 2021-12-13

**Authors:** Yong-Pei Wu, Shu-Mei Wang, Yu-Chi Chang, Chi Ho, Yu-Chia Hsu

**Affiliations:** 1 Department of Agronomy, Chiayi Agricultural Experiment Station, Taiwan Agricultural Research Institute, Chiayi 60014, Taiwan; wuypei@tari.gov.tw (Y.-P.W.); ycc71719@gmail.com (Y.-C.C.); chief5411@gmail.com (C.H.); 2 Department of Bio-Industry Communication and Development, National Taiwan University, Taipei 10617, Taiwan; wangsm@ntu.edu.tw; 3 Department of Agronomy, National Chiayi University, Chiayi 600355, Taiwan

**Keywords:** *japonica* DT3, drought tolerance, submergence tolerance, marker-assisted backcross, foreground selection, background selection

## Abstract

Flash flooding is a major environmental stressor affecting rice production worldwide. DT3 is a drought-tolerant, recurrent parent with a good yield, edible quality, and agronomic traits akin to those of an elite Taiwanese variety, Taiken9 (TK9). Progenies carrying *Sub1A* can enhance submergence stress tolerance and can be selected using the marker-assisted backcross (MAB) breeding method. For foreground selection, Sub1A and SubAB1 were utilized as markers on the BC_2_F_1_, BC_3_F_1_, and BC_3_F_2_ generations to select the submergence-tolerant gene, *Sub1A*. Background selection was performed in the *Sub1A*-BC_3_F_2_ genotypes, and the percentages of recurrent parent recovery within individuals ranged from 84.7–99.55%. BC_3_F_3_ genotypes (*N* = 100) were evaluated for agronomic traits, yield, and eating quality. Four of the eleven BC_3_F_4_ lines showed good yield, yield component, grain, and eating quality. Four BC_3_F_4_ lines, SU39, SU40, SU89, and SU92, exhibited desirable agronomic traits, including grain quality and palatability, consistent with those of DT3. These genotypes displayed a high survival rate between 92 and 96%, much better compared with DT3 with 64%, and demonstrated better drought tolerance compared to IR64 and IR96321-345-240. This study provides an efficient and precise MAB strategy for developing climate-resilient rice varieties with good grain quality for flood-prone regions.

## 1. Introduction

Rice (*Oryza sativa*) is one of the most important cereal crops grown worldwide and is widely cultivated in Asia. It provides approximately 50% of the calories for more than half of the world’s population. By 2050, the global population will reach 9.1 to 9.5 billion, 34 percent higher than that of today [1], and global rice consumption is estimated to increase to 650 million tons over time [2]. Nearly 640 million tons are grown in Asia, meeting the global requirement; however, climate change and heavy rainfall have affected global rice production and threaten long-term food security [3]. In recent years, the frequency of abnormal floods in Taiwan has increased substantially, particularly in Southeast Asian countries, such as Bangladesh, India, Indonesia, Nepal, and the Philippines [4]. In Bangladesh, 1.6 million hectares of rice fields are periodically affected by floods, while in India, about 32.2% of 16.1 million hectares of rice-growing fields are occasionally affected by floods [5,6]. Floods can cause up to a 100% reduction in rice yield, depending on the environmental and floodwater conditions [7]. Thus, the impact of catastrophic floods on rice production must be addressed urgently.

The *Sub1* QTL was mapped on chromosome 9, contributing to 70% of the phenotypic variation conferring an enhanced survival rate to rice under submergence [8]. It has been fine-mapped and positional cloned, and the cluster genes, including *Sub1A*, *Sub1B*, and *Sub1C*, were obtained in *indica* rice FR13A derivative [9,10] IR40931-26. The submergence-intolerant *japonica* rice Nipponbare contains *Sub1B* and *Sub1C* in the genome but lacks *Sub1A*, which encodes a putative DNA-binding protein with a single ERF/APETELA2 domain [10]. *Sub1A* repressed GA signal transduction in the submergence-tolerant rice under submergence stress, which affected the metabolic pathway of carbohydrates and the growth of FR13A for energy conservation, and rice growth resumed after the flood receded [11,12]. Thus, *Sub1* QTLs can be transferred into several different submergence-intolerant rice varieties via a conventional breeding program embedded with marker-assisted selection (MAS). In 2003, the International Rice Research Institute initiated a program and successfully introduced the *Sub1* QTL into six mega-varieties, Swarna, Samba Mahsuri, BR11, IR64, GR1009, and TDK1, using MAS [7,13,14,15,16]. Two high-yield potentials with submergence-tolerant rice varieties, BRRI dhan51 and BRRI dhan52, were released by the Bangladesh Rice Research Institute (BRRI) in 2010 [17]. Rice production is expected to surge in the submergence-prone areas of Bangladesh because the *Sub1* QTL increased the survival of submerged rice plants for a short duration.

Typhoons and heavy rainfall are the major causes of agriculture-related disasters in Taiwan. According to statistics from the Bureau of Meteorology, a total of 370 typhoons have hit Taiwan between 1911 and 2020. In the past hundred years, the annual average hit frequency of typhoons was 3–4 times. The number of typhoons has increased in Taiwan due to climate change in recent decades. The typhoon-related record-breaking increase in rainfall is a manifestation of global warming [18]. The heavy rain in 2021 has caused agricultural losses of TWD 150 million based on the statistics from the Council of Agriculture. Seedlings of rice of the second crop season in Taiwan are usually transplanted in August, the most vulnerable month to typhoons, which affects their survival rate. Introgression of *Sub1* QTLs in Taiwan *japonica* rice varieties is expected to enhance the survival rate of seedlings under submergence.

DT3, named Tainung83 in 2020, is an elite *japonica* rice line with good grain quality and drought tolerance. This variety does not contain the *Sub1A* gene; hence, it is susceptible to submergence stress. The introduction of *Sub1* QTL in IR96321-315-240, an *indica* rice of Swarna genetic background, conferred strong tolerance under submergence. This study was performed to convert DT3 into a submergence-tolerant genotype by introgressing *Sub1A* from IR96321-315-240, using the MAB breeding method. The study aimed to (i) develop DT3-*Sub1A* lines using MAB, (ii) evaluate the survival rate and related effects of DT3-*Sub1A* lines after submergence, and (iii) select good performance on agronomic traits and grain quality of individuals from the resulting submergence-tolerant lines. The development of submergence-tolerant lines can be a good genetic resource and provide new resilient rice cultivars for the future in Taiwan.

## 2. Results

### 2.1. Development of BC_3_F_6_ Submergence-Tolerant Lines Using Marker-Assisted Breeding

**Figure 1:**
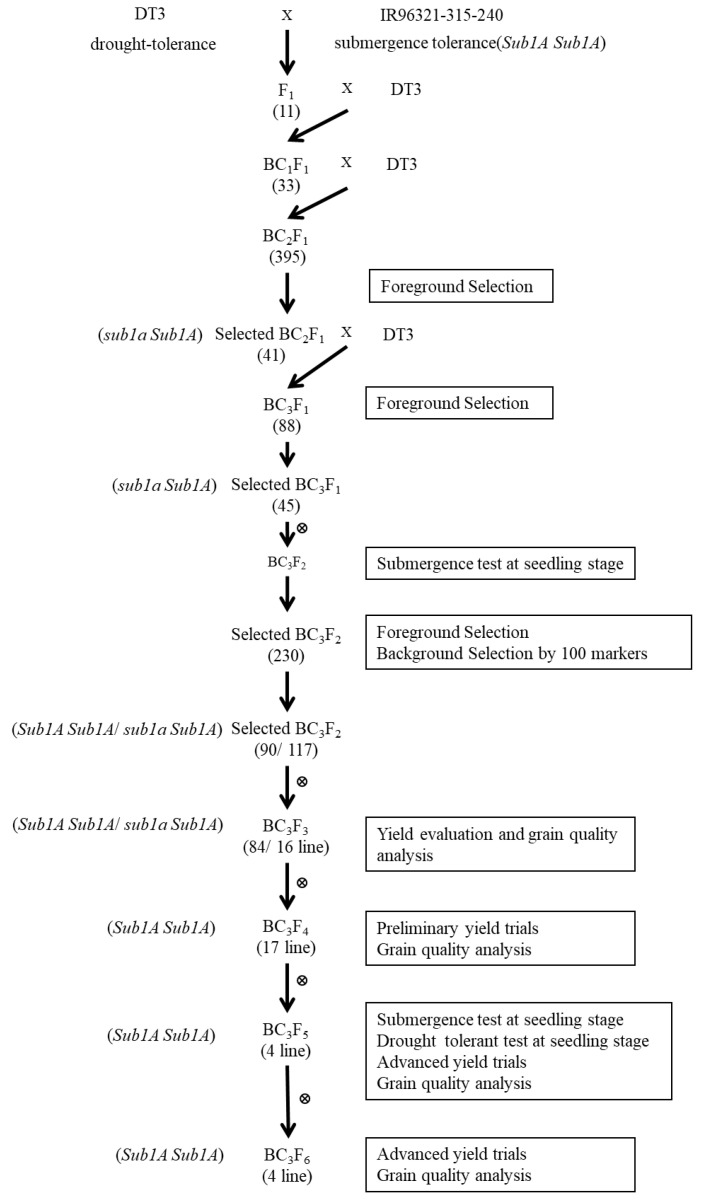
The schematic diagram for transferring *Sub1A* gene into Taiwanese *japonica* rice line, DT3, using marker-assisted backcrossing, detailing of submergence test and markers used for background selection. Numbers of plants selected in each generation are indicated in parentheses.

To develop a submergence-tolerant *japonica* cultivar, DT3 was used as a recurrent parent to backcross with IR96321-315-240 for three generations and then self-crossed to produce a BC_3_F_6_ population. The polymorphism was detected between the donor parent, IR96321-315-240, and recurrent parent DT3 with the markers Sub1A1 and Sub1AB1 for the *Sub1A* gene. In addition, 100 molecular markers (Figure S1), showing polymorphism between IR96321-315-240 and DT3, were used for background selection. During the breeding procedure, 33 BC_1_F_1_ plants were crossed with the recurrent parent DT3 (Figure 1). A total of 41 of 395 BC_2_F_1_ plants containing *Sub1A* were confirmed and selected by MAS. Functional marker selection was performed in three generations, including BC_2_F_1_, BC_3_F_1_, and BC_3_F_2_ generation. A total of 45 of the 88 BC_3_F_1_ were subjected to foreground selection.

**Figure 2:**
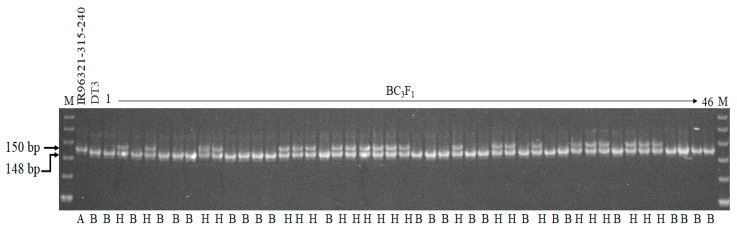
Partial view of the gel picture of the foreground selection with the marker Sub1AB1. M, 20 bp DNA ladder; 1–46, BC_3_F_1_ plants; A, homozygous donor allele; B, homozygous recurrent allele; H, heterozygous allele.

The 23 genotypes with “H” score are shown in the gel picture of foreground selection with marker Sub1AB1 (Figure 2). In the BC_3_F_2_, 800 plants were phenotyped at the seedling stage using submergence screening in the tank. A total of 230 plants were recovered after submergence treatment. In addition, surviving plants were selected for foreground and background selection. Out of 230 plants, 90 plants scored “A” (*Sub1A* homozygous), 117 plants scored “H” (*Sub1A*/*sub1a* heterozygous), and 23 plants scored “B” (*sub1a* homozygous) (Figure 1).

**Figure 3:**
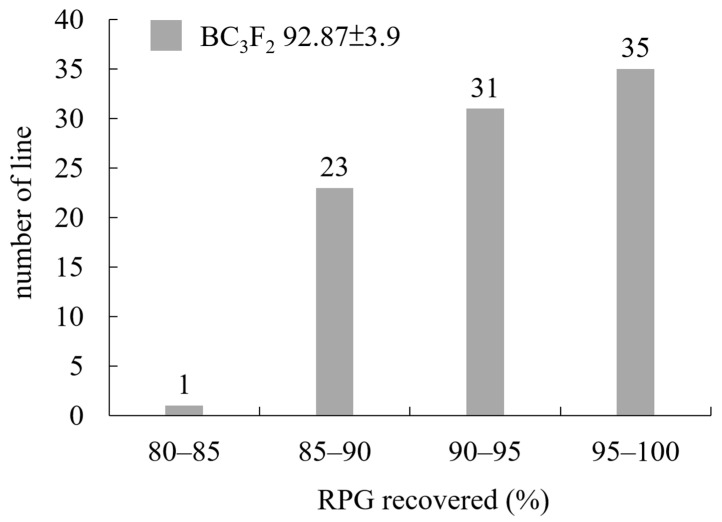
The frequency distribution of recurrent parent genome (RPG) recovered rate using marker-assisted backcrossing in BC_3_F_2_ population.

Background selection was conducted using 100 markers for 90 plants in the BC_3_F_2_ generation, which contained the *Sub1A* gene and possessed the recurrent genome content of DT3. The recovery of the recurrent parent genome ranged from 84.7 to 99.55%, with an average of 92.87% (Table S1). The highest percentage of recipient alleles was obtained in plant number 66 (99.55%), plant number 89 (98.5%), plant number 17 (98.4%), plant number 28 (98.1%), and plant number 77 (98%). Among them, 35 plants had a background recovery rate between 95 and 100%, 31 plants were 90–95%, 23 plants were 85–90%, and one plant was less than 85% (Figure 3). A total of 100 plants, 84 plants with homozygous *Sub1A* and 16 heterozygous *Sub1A* plants with good agronomic performance, were selected as BC_3_F_3_ lines (SU1-SU100) for further comparative yield tests.

### 2.2. Phenotyping and Evaluation of the Agronomic Performance of Newly Developed Sub1A Lines

One hundred BC_3_F_3_ rice plants were evaluated for their agronomic performance in the first crop season of 2019 at the Taiwan Agricultural Research Institute, Taiwan. The 28 lines were selected based on their agronomic traits, such as plant height, productive tiller number, heading uniformity, panicle type, and panicle weight. The yields among BC_3_F_3_ lines varied between 3667.4 kg ha^−1^ (SU33) and 9350 kg ha^−1^ (SU1) (Table S2). The rice palatability value for the BC_3_F_3_ lines varied from 59 to 75, with an average of 68.5; the highest and the lowest palatability were for SU48 and SU98, respectively. Seventeen BC_3_F_3_ lines were selected for preliminary yield trials based on the following threshold conditions: yield of more than 4000 kg ha^−1^, palatability value higher than 60, and percentage of chalky rice less than 40%.

Seventeen BC_3_F_4_ lines and their parents were evaluated for agronomic performance in the second crop season of 2019. In the range of agronomic traits of the BC_3_F_4_ lines, plant height varied between 93.4 and 120 cm, panicle number from 8 to 13, and grain yield from 4557.1 to 7857.1 kg ha^−1^ (Table S3). In addition, we classified the disease resistance reaction into five categories, resistance (R) to high sensitivity (HS), and evaluated the blast resistance of all the lines in the test nursery. The 17 BC_3_F_4_ lines displayed resistance (R) and moderate resistance (MR) to blast disease.

To evaluate the agronomic performance of these lines, 11 BC_3_F_4_ lines were selected for further investigation of the yield component and grain quality. The range of panicle weight varied from 3.56 to 4.96 g, spikelets per panicle from 148.56 to 185.1, percentage of seed set from 85.76 to 95.57%, and 1000-grain weight from 19 to 29.8 g (Table S4). In the grain quality analysis, the recurrent parent, DT3, had a recorded palatability value of 83.5, while the donor parent, IR96321-315-240, was 44. The rice palatability value for the BC_3_F_4_ lines differed from 72.5 to 87.5% (Table S5). Among them, the palatability values of SU39, SU40, and SU92 were higher than those of their parent line DT3 (83.5), which were 86, 87.5, and 84, respectively. After a comprehensive analysis of agronomic traits, yield, yield components, grain appearance, and eating quality, four lines (SU39, SU40, SU89, and SU92) met the following criteria: yield index higher than 90% (6647 kg ha^−1^) of the recurrent parent, percentage of chalky rice lower than 10%, or palatability value higher than 80, and were selected for the BC_3_F_5_ generation for advanced yield trials.

Four lines at the BC_3_F_5_ generation, and recurrent and donor parents, were evaluated in the first crop season of 2020. Significant differences were observed between the *Sub1A* lines and parental rice varieties for grain yield, panicle weight, and 1000-grain weight (Table 1). Table 1 shows that the selected line, SU92, has the potential for a higher yielding ability than those of IR96321-315-240 and DT3. In addition, three lines, SU39, SU89, and SU92, had significantly higher performance on 1000-grain weight compared to the parental lines. Grain quality is one of the goals of Taiwan’s breeding program. The mature rice grains of DT3 showed an 80.2% chalky rice rate, while the BC_3_F_5_ lines demonstrated a varied range of 33.3–86.5% with an average of 55.6%. There was no significant difference between SU39 and DT3 in the chalky rice rate, which was significantly lower than that of DT3 in three lines (SU40, SU89, and SU92), and SU89 was the lowest at 33.3% (Table 1).

The grain length of DT3 was 4.28 mm, those of BC_3_F_5_ lines were between 4.35 and 4.51 mm, which were significantly longer than that of DT3. The grain width of DT3 was 2.69 mm, and the BC_3_F_5_ lines were between 2.36 and 2.81 mm. Among them, SU39 and SU40 were significantly different from DT3 in grain length, while the other two lines, SU89 and 92, showed no difference.

We evaluated six physicochemical properties related to rice eating and cooking quality, including palatability and Rapid Visco Analyzer (RVA) parameters, in two parents and four BC_3_F_5_ lines planted in the first crop season. The palatability values of DT3 and BC_3_F_5_ lines were 58.6 and between 60.5 and 62.1, respectively. Except for SU89, the other three lines were not significantly different from the parental line DT3 (Table 2). In the analysis of the viscosity characteristics of rice grain, the peak viscosity (PKV) of SU92 was found to be 3551.25 cP, which was significantly higher than the 3159.5 cP of DT3. The PKV of three BC_3_F_5_ lines was between 3159.50 and 3404.25 cP and showed no significant difference compared to DT3 (Table 3). In addition, the breakdown viscosity (BDV) of SU92 was 2221 cP, which was significantly higher than that 1723.75 cP of DT3. The other three lines were between 1691.00–1882.75 cP, which was not significantly different from DT3.

### 2.3. Performance of the Sub1A Lines (BC_3_F_5_) under Submergence and Drought Conditions

**Figure 4:**
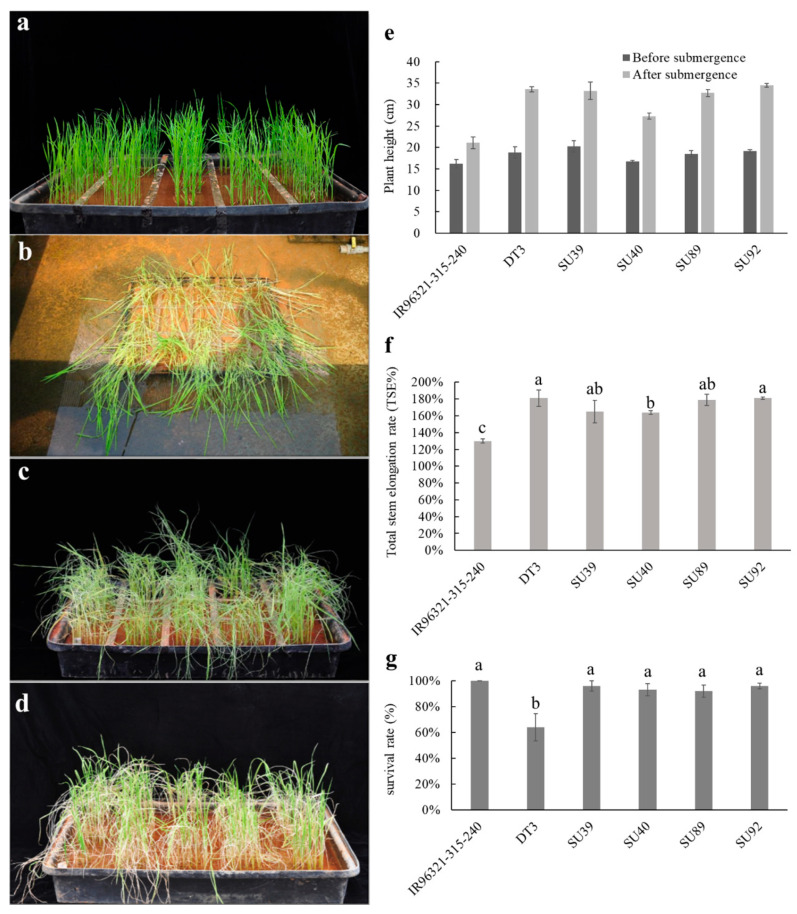
Characterization of BC_3_F_5_ lines and its parental lines, IR96321-315-240 and DT3, at rice seedling stage under submergences. (**a**) The phenotype of 21-day-old plants. (**b**) Phenotype of rice seedlings dewatering after 14 day submergence. (**c**) The phenotype of plants after 1 day of recovery from 14 day of submergence. (**d**) The phenotype of rice seedlings submerged for 14 day and then allowed to recover for 14 day. (**e**) Changes in plant height after submergence. (**f**) Total stem elongation rate (TSE%) of seedlings after 14 day submergence followed by 14 day recovery. (**g**) Survival rates of seedlings after 14 day submergence followed by 14 day recovery. Data are presented as the mean of three replications, and the bars represent the standard error of the mean (*n* = 3). Values with the different letters are significantly different (*p* < 0.05 by LSD test).

The 21-day-old seedlings of *Sub1A* lines were grown in plastic trays along with submergence-tolerant control IR96321-315-240 and susceptible control DT3 (Figure 4a). After 14 days of complete submergence, all lines showed leaves hanging down due to elongation once the water level dropped (Figure 4b). After one day of recovery from submergence treatment, some leaf tips appeared dry and withered (Figure 4c). However, after being submerged and recovering for 14 day, most of the plants grew fresh green leaves and recovered normal growth and development due to the presence of *Sub1A*-acquired submergence tolerance (Figure 4d). Seedlings of the submergence-tolerant parent IR96321-315–240 had an average elongation of 4.9 cm in plant height under 14 days of submergence and the total stem elongation rate (TSE%) of 130% (Figure 4e,f). The average elongation of DT3 was 14.8 cm in plant height, and the TSE% was 181%. In addition, the average elongation and TSE% of four *Sub1A* lines varied between 10.6 and 15.5 cm, and 164% and 181%, respectively (Figure 4e,f). Except for the lower survival rate (an average of 64%) of susceptible parent DT3, the other *Sub1A* lines had a similar higher survival rate to IR96321-315-240 (Figure 4g).

**Figure 5:**
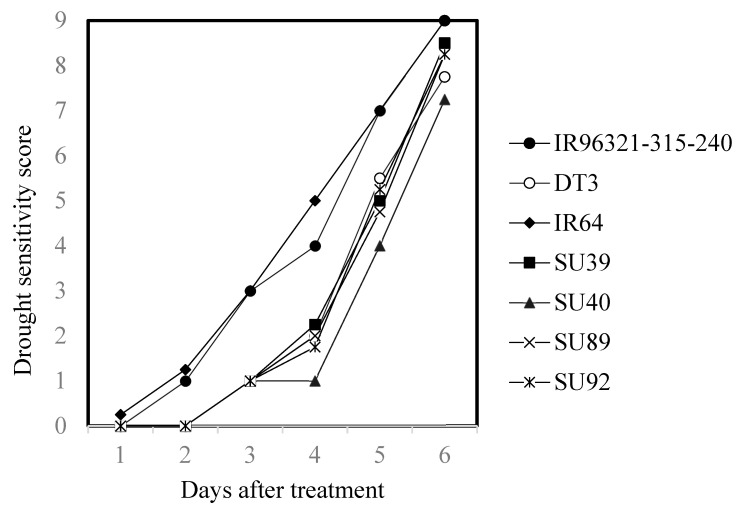
The drought sensitivity score of drought-tolerant donor parent IR96321-315-240, drought-susceptible variety IR64, recurrent parent DT3 and the 4 BC_3_F_5_ backcrossing line under the 28% PEG-6000 osmotic solution.

The recurrent parent DT3 was used in this study selected from a crossing combination between Taikeng9 and the drought-tolerant cultivar Hang-yu15 [19]. Four *Sub1A* lines and the parental lines were used to evaluate the drought tolerance under 28% PEG-6000 simulated drought stress. Five days after osmotic stress in the seedling stage, two control cultivars IR96321-315-240 and IR64, were grouped on scale 7 of drought sensitivity, but all the *Sub1A* lines had a varied scale between 4 and 5.25 similar to that of recurrent parent DT3 (Table 4). According to the daily scale trend chart of drought sensitivity, for all the rice varieties (lines) under 28% PEG-6000 drought stress, the performance of the four *Sub1A* lines was significantly different from that of the control varieties under stress (Figure 5, Table 4).

## 3. Discussion

In addition to the second crop season of rice planting in Taiwan being affected by typhoons, the first crop season faces water shortage crises as well, which affect rice production. Taiwan has experienced a serious water shortage since late 2020. It was also the first time in the last 56 years, and no typhoons passed through Taiwan. According to the Council of Agriculture statistics, a total of 24 percent of planted areas were affected by drought in Taiwan, which evaluated the irrigation of 74,000 hectares of first crop rice in 2021. Therefore, the development of submergence and drought-tolerant varieties can overcome the Taiwan rice production crisis in the future. DT3 is described as a *japonica* rice line with a high yield potential (6–7 t/ha), excellent grain quality, and good drought tolerance [19]. As an extremely valuable yet submergence-susceptible line, DT3 was selected as the focus of this study to increase the submergence tolerance through the introgression of *Sub1A*.

Typical flash flooding results in rapidly rising water levels with submergence for 1–2 weeks. Complete submergence results in accelerated energy consumption in rice seedlings and affects plant growth. It is prone to lodging after the water level recedes [20,21]. To date, FR13A has been recognized as the most submergence-tolerant cultivar and is therefore widely used in rice breeding programs for submergence tolerance [7,22,23,24,25]. Rice breeding was accelerated following the identification of QTL *Sub1* on chromosome 9 and understanding the mechanism of gene regulation [10,26,27]. QTL *Sub1* accounts for up to 70% of the submergence tolerance. The cluster genes, *Sub1A*, *Sub1B*, and *Sub1C*, are the different genes that were related to ethylene response factor (ERF)-like genes at the *Sub1* locus. Overexpression of a *Sub1A-1* full-length cDNA in a submergence-intolerant *japonica* rice conferred enhanced tolerance to the plants, which demonstrates that *Sub1A*-*1* is a primary determinant of submergence tolerance [10]. The 76 rice genotypes from the International Rice Germplasm Collection surveyed for *Sub1* specific markers, Sub1A and Sub1C, indicated that all accessions without the *Sub1A* gene were submergence intolerant. In the gene expression analyses, the results also demonstrate *Sub1C* expression did not associate with submergence tolerance [28]. In this study, the backcross population was constructed using the Swarna-*Sub1* near-isogenic line IR96321-315-240 as a submergence donor parent crossed with good grain quality and drought-tolerant line DT3.

Backcrossing is a conventional method of transferring one or more genes of interest from a donor parent into an elite variety. However, it cannot accurately select the target trait based on the phenotype that is controlled by a specific gene during each round of backcrossing. Modified backcrosses combined with a molecular-marker-assisted selection have already been demonstrated to improve the efficiency of plant breeding, leading to the development of genetic resources and the precise development of tolerance [7,29,30,31,32]. In this study, we successfully developed lines demonstrating submergence tolerance in the background selection of DT3 without altering the main features of the recipient variety. In addition, we accelerated the efficiency of the breeding program using gene-specific markers of *Sub1A* for foreground selection at BC_2_F_1_, BC_3_F_1_, and BC_3_F_2_ (Figure 1). MAB is considered more cost-effective and reduces the use of labor and resources compared to the classical backcross breeding approach. The modified MAB in our study used large population sizes (395 plants) for foreground selection in the BC_2_F_1_ generation. Foreground selection in BC_1_F_1_ was not validated, but 41 plants with the target gene were sufficient for the next-generation selection. The desired traits that were lacking in Taiwan’s popular rice varieties can be improved in less time and with more precision using the results of our study.

In the BC_3_F_2_, a total of 230 plants were recovered from 800 plants after submergence treatment. Among them, 23 plants were shown to be without the Sub1A gene by marker analysis. The plants without *Sub1A* can survive, and we speculate that there are two possible reasons: (1) Although the *Sub1* QTL accounts for about 70% of the phenotype variation of the submergence tolerance [8], 30% of the phenotypic variation is controlled by other loci. Therefore, surviving plants cannot be ruled out as being affected by other loci. (2) Submergence is abiotic stress. Plants develop various physiological and biochemical mechanisms to adapt to stress, including the development of aerenchyma and adventitious roots for improved aeration, activation of internode and petiole elongation to outgrow submergence water, and conservation of energy until floodwater subsides [33,34,35].

High-density molecular markers used for background selection can greatly accelerate the recovery rate of the recurrent parental background. For DT3, an average of eight markers were used per chromosome, and the average distance between the two markers was 14.55 cm. In the BC_3_F_2_ generation, 800 plants were completely submerged for 14 days, and the surviving plants were subjected to foreground selection. A total of 207 BC_3_F_2_ plants that were *Sub1* homozygous or *Sub1*/*sub1* heterozygous were selected for background selection to evaluate the recovery rate. The best plant contained 99.6% of the recipient genome. The range of recovery rate was between 84.7 and 99.6%, with an average of 92.9% (Figure 3). Although we did not routinely use the background selection in each generation, the recovery rate was lower than that of the general MAB strategy [7]. The modified MAB in this study can therefore enhance the efficiency of breeding selection.

The development of rice varieties with submergence tolerance, high grain yield potential, and good grain and cooking quality can be immediately useful for flood-prone areas and help farmers increase their production and income [32]. Several lines exhibiting submergence-tolerance and jasmine-like cooking quality with low amylose have been developed in Thailand [30]. In this study, the agronomic performance evaluation of BC_3_F_4_ to BC_3_F_5_, a few elite lines were obtained and had important features, such as the genetic background of DT3. All the lines for most of the agronomical traits were, in general, like the recipient parent DT3. However, three candidate lines, SU40, 89, and 92, showed good grain yield and palatability (Table 1 and Table 2), which was further confirmed by the multi-location evaluation. In addition, in previous studies, high taste quality was associated with a high peak viscosity and breakdown, and low setback viscosity, while white rice with low cold paste viscosity has a softer taste than cold rice [36,37]. In this study, SU92 had the highest peak and breakdown viscosity and the lowest cold paste viscosity. Therefore, SU92 may have better taste quality and cold rice quality than the parental line DT3 (Table 3).

Despite having gas spaces (aerenchyma) form as an adaptation to submergence, many lowland rice cultivars are still sensitive to complete submergence. If leaves of seedlings elongate their shoots rapidly to contact the aerial interface, they can escape the stress and successfully promote survival. However, their elongation growth can exhaust energy reserves and cause death during complete submergence [5,38,39]. In this study, donor parent IR96321-315-240 with the *Sub1A* gene caused it to enter into a “quiescent” state under submergence, and shoot elongation was no significant difference compared to normal condition (Figure 4). In addition, the donor parent and four lines with *Sub1A* introgression had a significantly higher survival rate compared to the recipient parent, DT3. In addition, we found that DT3 was markedly elongated compared to IR96321-315-240 and lodged severely after submergence. Although the survival rate of DT3 was lower than that of IR96321-315-240 and its backcross offspring after 14 days of complete submergence, its survival ability remained substantial, which coincided with previous findings of studies on upland rice [40].

Differences in the drought tolerance of plants can be evaluated by the visual scoring of leaf rolling, drying, and wiling symptoms, reflecting the dehydration status via simulated drought stress treatments of PEG [24,41]. In this study, the control, parent lines, and *Sub1A* lines were evaluated for their ability to tolerate dehydration in response to PEG treatment. IR64 and IR96321-315-240 exhibited a score of 7 after 5 days of 28% PEG treatment, suggesting two varieties are highly susceptible genotypes, while DT3 and *Sub1A* lines recorded a score of 4–5.5, and were moderately tolerant (Table 4 and Figure 5). In general, *Sub1A* lines had the successful recovery of good grain and eating quality from DT3 with improved tolerance against submergence and drought. This is a significant achievement that will provide farmers with new varieties for cultivation.

## 4. Materials and Methods

### 4.1. Plant Materials

The experiments were carried out to transfer the *Sub1* locus into DT3, an elite *japonica* line with drought tolerance, lodging tolerance, blast resistance, and good grain quality [19]. This line was derived from the cross TaiKen No. 9 (TK9, *Oryza sativa* ssp. *japonica*) and Huhan 15 (*Oryza sativa* ssp. *indica*). The yield potential of this line is 6.5 tons ha^−1^. The submergence-tolerant rice genotype IR96321-315-240 was used as the *Sub1* donor parent. A cross was made between DT3 and IR96321-315-240, with F1 plants backcrossed thrice with DT3 to obtain BC_3_F_1_ plants, which were self-crossed to obtain the BC_3_F_6_ progeny. Selections based on foreground, background, and submergence tests were performed from BC_2_F_1_ to BC_3_F_2_ as a means of identifying lines similar to those of the recurrent parent.

### 4.2. Evaluation of Sub1A Lines for Submergence Tolerance

Before the submerged stress treatment, seeds were surface-disinfected with the recommended dose of the benomyl wettable powder (Fulon Chemical Industrial Co., Ltd.; Taoyuan City, Taiwan), which was diluted 1000 times for one day, and seeds were germinated in Petri plates. Germinated seedlings were transferred to seedling trays with soil and grown for up to 21 days. Seedlings were subjected to submergence stress by keeping the pots inside 1 m tanks filled with water. A randomized complete block design was used under the stress condition, with three reps and 25 plants for each replicate of each line. Plants were removed after two weeks of submergence treatment. The percentage of survival was assessed 14 days after de-submergence in the greenhouse.

### 4.3. Evaluation of Sub1A Lines for Osmotic Stress Tolerance

Seeds of DT3, IR64, and 4 BC_3_F_5_ lines were surface-disinfected with benomyl and germinated in plates. Germinated seeds were put in 96-well hydroponic trays and grown for up to 21 days under hydroponic conditions (Yoshida solution, Chiayi, Taiwan) [42]. Rice seedlings were stressed in a Yoshida solution containing 28% polyethylene glycol (PEG) 6000. Plants were scored for their responses to osmotic stress every day using a standard evaluation system [43].

### 4.4. Evaluation of Sub1A Lines for Blast Resistance

The rice blast nursery was conducted in an upland field in the second season of 2019. Natural *Magnaporthe grisea* infection was favored at the nursery by a high level of nitrogen fertilization and the planting of the susceptible rice cultivar ‘Lomello’, and resistant *japonica* rice cultivars ‘Tainung No. 70’ and *indica* rice ‘Taichung Sen No. 10’ in the field. In each replication, 5 g seeds for each rice variety (line) were planted in a row (50 cm row length with 10 cm spacing). Evaluation of leaf symptoms was performed on the leaves of every plant in a row using a standard visual scale (0–9) developed by the International Rice Research Institute [40]. The lines that had an average grade of 1–3, 3–5, 5–6, 6–7, and >7 were regarded as resistant (R), moderately resistant (MR), moderately susceptible (MS), susceptible (S), and highly susceptible (HS), respectively. The resistance reaction of each plant was scored from the two replications and averaged to represent the severity of leaf blast on each rice variety (line).

### 4.5. Evaluation of Agronomic Traits

During the first and second crop seasons of 2019, the thirty-day-old seedlings of the BC_3_F_3_ and BC_3_F_4_ lines and both parents were transplanted into three rows, with 24 plants per row, per entry, at 15 × 25 cm spacing, at the Chiayi Agricultural Experiment Station Farm of the Taiwan Agricultural Research Institute. The agronomic performance, grain quality, and palatability of the 28 BC_3_F_3_ and 11 BC_3_F_4_ lines were measured. In BC_3_F_5_, field planting followed a randomized complete block design with four replications (blocks). Four lines were selected, and two parents were transplanted into the plots. Each plot consisted of three rows with 20 plants per row at 30 × 15 cm. The following agronomic traits were recorded for each line: number of grains per panicle, panicle length (cm), panicle weight (g), spikelets/panicles, seed set (%), 1000-seed weight (g), grain yield (kg ha^−1^), and yield index (%). In addition, the grain quality, including percentage of chalky rice (%), seed length (mm), seed width (mm), seed length-width ratio, seed thickness (mm), and palatability, were also investigated and analyzed. For palatability analyses, approximately 33 g of rice flour was hulled and ground into fine flour for the palatability evaluation, which was performed using a palatability analyzer system (Toyo Taste Meter, Model MA-30). The sample processing method was carried out in accordance with the manufacturer’s operation manual (TRCM Co., Toyo Rice Polishing Machine Factory, Osaka, Japan), as previously described [44].

In addition, for the viscosity analysis of cooked rice grain, approximately 3 g rice flour was mixed with 25 mL water and used for RVA profile evaluation, which was performed using a Rapid Visco Analyzer (Model No. RVA-4, Newport Scientific, Sydney, Australia), according to the Standard Method AACC61-02 released by the American Association of Cereal Chemists. The sequential temperature curve for a 12.5 min test was as follows: (1) incubation at 50 °C for 1 min; (2) increased to 95 °C and held for 2.5 min; (3) cooling to 50 °C and held at 50 °C until the end. The RVA profiles were characterized by five parameters: peak viscosity (PKV), hot paste viscosity (HPV), breakdown viscosity (BDV = PKV − HPV), cool paste viscosity (CPV), and setback viscosity (SBV = CPV − PKV). Statistical analysis was performed with independent samples using least significant difference (LSD).

### 4.6. DNA Isolation and PCR Amplification

Rice genomic DNA extraction, with modifications, was adopted for mini preparation [44]. Approximately 5 cm of fresh leaf tissue from seedlings was homogenized with 300 μL extraction buffer (100 mm Tris-HCl, pH 9.0; 40 mm EDTA-2Na, pH 8.0; 1.67% SDS) at 30 strokes/s for 2 min using a TissueLyser (Qiagen Retsch GmbH & Co. KG, Haan, Germany). A total of 150 μL of benzyl chloride was added to the homogenized tissue and vortexed. After incubation in a 50 °C water bath for 15 min, 150 μL of 3 M sodium acetate (pH 5.2) was added. Supernatants were centrifuged at 15,000 rpm for 15 min at 4 °C, and 300 μL ice-cold isopropanol was added to precipitate DNA. After centrifugation at 15,000 rpm for 10 min, DNA pellets were saved and washed with 70% ethanol, air-dried, and dissolved in 50 μL TE buffer. A 10 μL PCR reaction containing 10 ng genomic DNA, 2.5 μM forward and reverse primers, and 5 μL Multiplex PCR Master Mix (QIAGEN, Inc., Redwood City, CA, USA) was performed using a thermocycler (GeneAmp PCR System 9700, Life Technologies Corp., Carlsbad, CA, USA) at 94 °C for 2 min for 1 cycle; 94 °C for 30 s, 55 °C for 20 s, 72 °C for 30 s for 35 cycles, and 72 °C for 2 min for 1 cycle. Following PCR, 1 μL of amplified DNA products was separated by 6% polyacrylamide gel in 0.5× TBE at 100 v (Dual Triple-Wide Mini-Vertical System, C. B. S. Scientific, San Diego, CA, USA) for 60 min.

### 4.7. Marker Analysis

Foreground selection was performed using functional and linkage markers, Sub1A1 and Sub1AB1 (Table S6) [15]. In addition, a total of 100 markers, including 66 SSRs, 8 STS, and 26 InDel, distributed evenly on the 12 chromosomes, were used for genotyping in BC_3_F_2_ with an average marker interval of 14.55 cm, and were used in a genome-wide survey to identify the chromosome segment substitution locations. These polymorphic markers were used for background selection to select plants with maximum recovery of the recurrent parent genome. The genotypes from polymorphic bands were recorded as A (IR96321-315-240), B (DT3), and H (DT3/IR96321-315-240). The Graphical Geno Types Version 2.0 [45] software program was used for the assessment of the recurrent parent genome (%RPG) in the selected recombinants, based on marker data.

## 5. Conclusions

Compared to conventional backcrossing, marker-assisted backcross breeding is an effective and reliable approach for transferring the *Sub1A* gene in rice. In this study, we constructed a low-cost, low-input, and high-efficiency molecular-marker-assisted selection platform for rice breeding programs. By using a few foreground selections, phenotyping of submergence tolerance, and background selection at the beginning of the breeding process, while combining the evaluation of agronomic traits in late generations, we successfully developed several submergence- and drought-tolerant lines, with high yield and good grain and eating quality. These *Sub1A* lines with a DT3 genetic background are expected to have a high impact on domestic rice production stability and reduce the risk of rice yield loss in flood-prone rain-fed areas caused by climate change.

## Figures and Tables

**Table 1:** The grain yield evaluation and yield components of IR96321-315-240, DT3 and 4 BC_3_F_5_ backcross lines in advanced yield trial at the first cropping season in 2020.

Line or Variety	Grain Yield Evaluation		Yield Components					
	Grain Yield (kg ha^−1^)	Yield Index ^z^ (%)	Panicle Number (No.)	Panicle Length (cm)	Panicle Weight (g)	Spikelets per Panicle (No.)	Seed Set (%)	1000-Grain Weight (g)
IR96321-315-240	4617 ^b^	73.7	19.8	19.0	2.08 ^c^	117.0	84.7	19.0 ^c^
DT3	6309 ^ab^	100.0	12.8	19.8	2.72 ^b^	138.3	79.6	22.3 ^b^
SU39	5853 ^ab^	95.9	14.2	18.9	3.39 ^a^	150.4	80.4	25.9 ^a^
SU40	6168 ^ab^	101.8	14.4	20.4	2.88 ^ab^	154.5	84.5	20.9 ^b^
SU89	6101 ^ab^	97.8	14.1	19.5	3.14 ^ab^	143.2	84.2	24.7 ^a^
SU92	7345 ^a^	114.6	15.1	19.7	3.01 ^ab^	133.6	85.0	25.3 ^a^

Means followed by the different letters are significantly different at the 5% level by least significant difference (LSD) test. ^z^ The yield indices are compared with that of DT3.

**Table 2:** The seed appearance and palatability of IR96321-315-240, DT3 and 4 BC3F5 backcross lines in advanced yield trial at the first cropping season in 2020.

Line or Variety	Seed Appearance					Palatability
	Chalky Rice (%)	Seed Length (mm)	Seed Width (mm)	Seed Length-Width Ratio	Seed Thickness (mm)	
IR96321-315-240	53.1 ^bc^	4.69 ^a^	2.11 ^d^	2.23 ^a^	1.81 ^d^	35.5 ^c^
DT3	80.2 ^a^	4.28 ^d^	2.69 ^b^	1.59 ^d^	2.04 ^a^	58.6 ^b^
SU39	86.5 ^a^	4.38 ^c^	2.81 ^a^	1.56 ^e^	2.03 ^a^	61.4 ^ab^
SU40	59.2 ^b^	4.35 ^c^	2.36 ^c^	1.85 ^b^	1.90 ^c^	61.9 ^ab^
SU89	33.3 ^d^	4.51 ^b^	2.70 ^b^	1.67 ^c^	2.03 ^a^	62.1 ^a^
SU92	43.3 ^c^	4.51 ^b^	2.69 ^b^	1.67 ^c^	2.00 ^b^	60.5 ^ab^

Means followed by the different letters are significantly different at 5% level by least significant difference (LSD) test.

**Table 3:** Viscograph pasting of IR96321-315-240, DT3 and 4 BC_3_F_5_ backcross lines in advanced yield trial at the first cropping season in 2020.

Line or Variety	Peak Viscosity (PKV)	Hot Paste Viscosity (HPV)	Breakdown Viscosity (BDV)	Cool Paste Viscosity (CPV)	Setback Viscosity (CSV)
IR96321-315-240	2350.33 ^c^	1463.33 ^b^	887.00 ^c^	3470.33 ^a^	2007.00 ^a^
DT3	3159.50 ^b^	1435.75 ^b^	1723.75 ^b^	2302.25 ^b^	866.50 ^b^
SU39	3197.75 ^b^	1506.75 ^a^	1691.00 ^b^	2414.25 ^b^	907.50 ^b^
SU40	3229.25 ^b^	1393.75 ^b^	1835.50 ^b^	2228.50 ^b^	834.75 ^b^
SU89	3404.25 ^ab^	1521.50 ^a^	1882.75 ^b^	2424.00 ^b^	902.50 ^b^
SU92	3551.25 ^a^	1330.25 ^b^	2221.00 ^a^	2200.50 ^b^	870.25 ^b^

Means followed by the different letters are significantly different at 5% level by least significant difference (LSD) test.

**Table 4:** The scale of drought tolerance among IR96321-315-240, DT3 and 4 BC_3_F_5_ backcross lines under the treatment of 28% PEG 6000 solution at seedling stage.

Line or Variety	Days after Treatment					
	1	2	3	4	5	6
IR96321-315-240	0.00 ^b^	1.00 ^a^	3 ^a^	4.00 ^a^	7.00 ^a^	9.00 ^a^
DT3	0.00 ^b^	0.00 ^b^	1 ^b^	2.00 ^b^	5.50 ^b^	7.75 ^ab^
IR64 ^z^	0.25 ^a^	1.25 ^a^	3 ^a^	5.00 ^a^	7.00 ^a^	9.00 ^a^
SU39	0.00 ^b^	0.00 ^b^	1 ^b^	2.25 ^b^	5.00 ^b^	8.50 ^ab^
SU40	0.00 ^b^	0.00 ^b^	1 ^b^	1.00 ^b^	4.00 ^c^	7.25 ^b^
SU89	0.00 ^b^	0.00 ^b^	1 ^b^	2.00 ^b^	4.75 ^bc^	8.25 ^ab^
SU92	0.00 ^b^	0.00 ^b^	1 ^b^	1.75 ^b^	5.25 ^b^	8.25 ^ab^

Means followed by different letters are significantly different at the 5% level as calculated by the least significant difference (LSD) test. ^z^ The sensitive control of drought experiment.

## Supplementary Materials

The following are available online at https://www.mdpi.com/article/10.3390/ijms222413365/s1.
