# Supplementary material for: Submergence Gene Sub1A Transfer into Drought-Tolerant japonica Rice DT3 Using Marker-Assisted Selection
Source: Int J Mol Sci. 2021 Dec 13;22(24):13365. doi: 10.3390/ijms222413365 (PMC8705020; doi:10.3390/ijms222413365)
Supplement: Supplementary file 1 [file ijms-22-13365-s001.zip › ijms-1480418-supplementary.pdf]

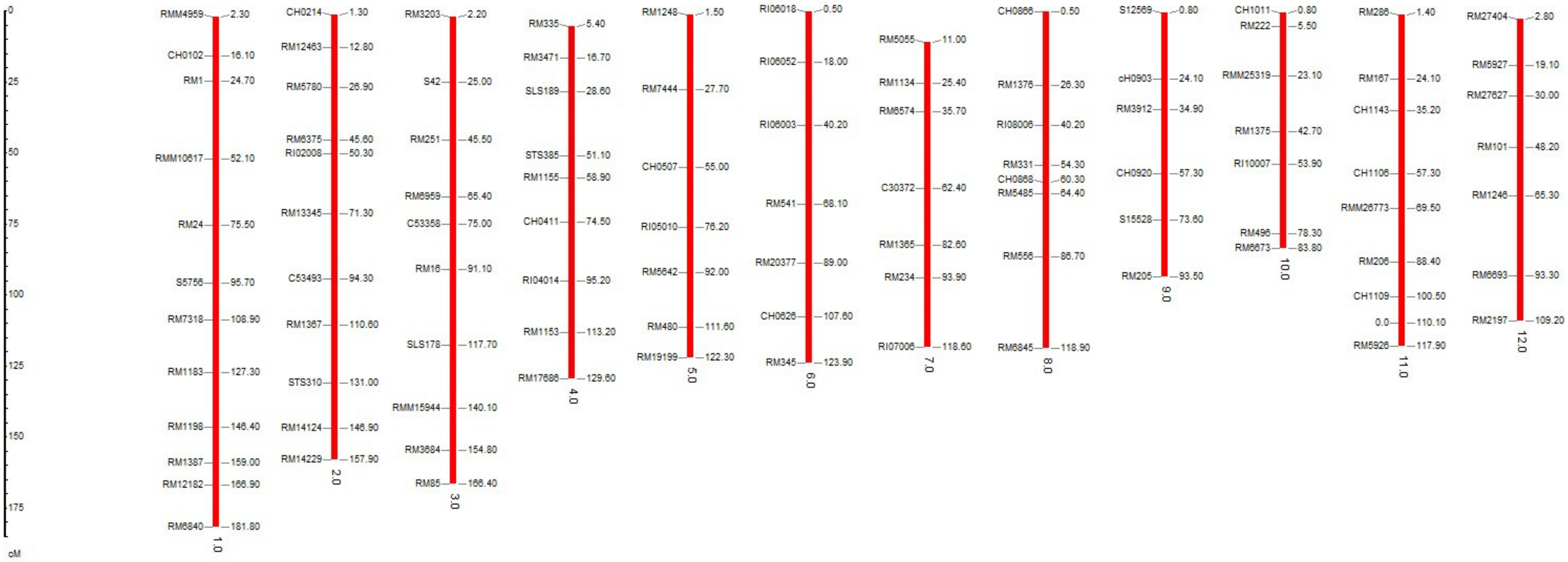

Figure S1. The 100 polymorphic markers used for background selection. The prefixes RM indicates SSR markers; C, E and S indicate STS markers; CH, STS, SLS and RI indicate indel markers.

Table S1. The genome composition of BC3F2 derived from DT3/IR96321-315-240 using 100 polymorphic markers used for marker-assisted selection (MAS).

Individuals[z]	A (%)[y]	B (%)[x]	H (%)[w]	N (%)[v]	RPG recovered (%)	Total (cM)	Recombinant[u]	H-segments[t]
DT3	0.0	100.0	0.0	0.0	100.00	1493.3	0	0
1	9.2	81.9	8.9	0.0	86.35	1493.3	16	6
2	0.9	93.8	5.3	0.0	96.45	1493.3	5	3
3	1.3	93.6	5.1	0.0	96.15	1493.3	5	3
4	7.9	86.0	6.2	0.0	89.10	1493.3	17	6
5	6.2	82.5	11.2	0.0	88.10	1493.3	16	6
6	0.9	93.9	5.2	0.0	96.50	1493.3	6	3
7	2.6	85.7	11.7	0.0	91.55	1493.3	11	5
8	3.8	90.6	5.6	0.0	93.40	1493.3	8	2
9	0.9	95.6	3.5	0.0	97.35	1493.3	3	1
10	7.3	85.2	7.5	0.0	88.95	1493.3	18	6
11	2.3	89.5	8.3	0.0	93.65	1493.3	7	3
12	2.7	94.6	2.8	0.0	96.00	1493.3	8	2
13	3.9	87.0	9.1	0.0	91.55	1493.3	8	4
14	2.7	85.1	12.2	0.0	91.20	1493.3	11	5
15	5.7	84.8	9.5	0.0	89.55	1493.3	12	5
16	1.2	96.1	2.7	0.0	97.45	1493.3	5	2
17	0.0	96.8	3.2	0.0	98.40	1493.3	3	2
18	7.1	83.0	9.9	0.0	87.95	1493.3	12	3
19	0.5	94.8	4.6	0.0	97.10	1493.3	6	3
20	4.1	92.7	3.1	0.0	94.25	1493.3	8	3
21	1.6	90.2	8.2	0.0	94.30	1493.3	9	4
22	2.1	94.0	3.9	0.0	95.95	1493.3	7	2
23	1.4	90.7	7.9	0.0	94.65	1493.3	10	4
24	2.2	95.1	2.7	0.0	96.45	1493.3	9	3
25	0.4	95.1	4.6	0.0	97.40	1493.3	7	3
26	2.3	94.8	2.9	0.0	96.25	1493.3	6	1
27	1.9	95.2	2.9	0.0	96.65	1493.3	8	3
28	1.3	97.5	1.2	0.0	98.10	1493.3	5	2
29	10.8	83.2	6.1	0.0	86.25	1493.3	16	4
30	1.0	95.2	3.8	0.0	97.10	1493.3	5	2
31	6.2	93.2	0.5	0.0	93.45	1493.3	4	1
32	1.4	93.4	5.1	0.0	95.95	1493.3	10	4
33	1.4	95.7	2.8	0.0	97.10	1493.3	6	2
34	8.6	84.0	7.4	0.0	87.70	1493.3	9	3
35	1.6	96.2	2.2	0.0	97.30	1493.3	8	3
36	3.9	93.9	2.2	0.0	95.00	1493.3	8	2
37	0.5	94.5	5.0	0.0	97.00	1493.3	5	2
38	1.8	96.8	1.4	0.0	97.50	1493.3	6	2
39	9.9	84.7	5.4	0.0	87.40	1493.3	14	3
40	2.3	87.6	10.1	0.0	92.65	1493.3	14	6
41	5.0	88.9	6.1	0.0	91.95	1493.3	9	3

42	5.8	89.9	4.3	0.0	92.05	1493.3	13	4
43	3.5	83.4	13.1	0.0	89.95	1493.3	13	6
44	11.5	82.4	6.1	0.0	85.45	1493.3	14	3
45	2.6	89.8	7.6	0.0	93.60	1493.3	11	4
46	5.1	84.4	10.5	0.0	89.65	1493.3	15	5
47	10.6	81.6	7.8	0.0	85.50	1493.3	15	4
48	7.3	89.0	3.7	0.0	90.85	1493.3	10	3
49	3.3	96.2	0.5	0.0	96.45	1493.3	4	1
50	7.1	87.4	5.6	0.0	90.20	1493.3	8	2
51	1.0	93.9	5.0	0.0	96.40	1493.3	7	3
52	7.3	84.7	8.0	0.0	88.70	1493.3	18	6
53	4.8	90.4	4.8	0.0	92.80	1493.3	9	4
54	2.7	93.2	4.1	0.0	95.25	1493.3	8	2
55	3.8	88.7	7.5	0.0	92.45	1493.3	6	3
56	5.1	86.3	8.6	0.0	90.60	1493.3	14	5
57	3.3	88.5	8.2	0.0	92.60	1493.3	12	4
58	1.4	95.3	3.3	0.0	96.95	1493.3	6	2
59	10.8	84.9	4.3	0.0	87.05	1493.3	11	3
60	1.1	95.5	3.4	0.0	97.20	1493.3	4	1
61	0.0	99.1	0.9	0.0	99.55	1493.3	3	2
62	1.3	93.8	5.0	0.0	96.30	1493.3	4	2
63	2.0	96.7	1.2	0.0	97.30	1493.3	4	1
64	5.9	93.2	0.9	0.0	93.65	1493.3	5	1
65	5.4	88.0	6.6	0.0	91.30	1493.3	9	3
66	6.2	81.9	11.9	0.0	87.85	1493.3	18	6
67	5.7	84.5	9.8	0.0	89.40	1493.3	14	5
68	8.8	84.1	7.1	0.0	87.65	1493.3	15	4
69	1.3	96.2	2.5	0.0	97.45	1493.3	4	1
70	3.3	88.4	8.3	0.0	92.55	1493.3	13	5
71	6.3	85.7	8.0	0.0	89.70	1493.3	15	5
72	4.1	87.9	8.0	0.0	91.90	1493.3	11	4
73	4.7	92.5	2.8	0.0	93.90	1493.3	6	2
74	0.0	95.5	4.5	0.0	97.75	1493.3	6	3
75	2.7	93.4	3.9	0.0	95.35	1493.3	7	3
76	1.2	94.7	4.1	0.0	96.75	1493.3	4	2
77	0.0	96.0	4.0	0.0	98.00	1493.3	5	3
78	5.3	82.9	11.8	0.0	88.80	1493.3	17	5
79	5.5	93.2	1.3	0.0	93.85	1493.3	5	1
80	5.5	85.4	9.1	0.0	89.95	1493.3	11	3
81	3.5	86.3	10.2	0.0	91.40	1493.3	13	4
82	8.5	83.1	8.4	0.0	87.30	1493.3	12	4
83	5.9	85.8	8.3	0.0	89.95	1493.3	14	4
84	7.8	82.3	9.9	0.0	87.25	1493.3	16	5
85	11.5	80.9	7.6	0.0	84.70	1493.3	15	4
86	8.0	84.7	7.3	0.0	88.35	1493.3	10	2
87	3.9	87.7	8.4	0.0	91.90	1493.3	11	3
88	1.6	87.1	11.2	0.0	92.70	1493.3	14	7
89	0.7	97.7	1.6	0.0	98.50	1493.3	4	1
90	8.1	81.3	10.6	0.0	86.60	1493.3	16	6
IR69321	100.0	0.0	0.0	0.0	0.00	1493.3	0	0

[z] The arrangement was based on the percentage of genetic similarity with DT3.

[y] The percent of genetic similarity with IR96321-315-240 genome.

[x] The percent of genetic similarity with DT3 genome.

[w] The percent of heterozygote.

[v] The percent of missing data.

[u] The number of chromosome recombination.

[t] The number of heterozygous segments in sample.

Table S2. The grain yield evaluation, seed appearance, and palatability of 28 selected BC_3F_3 backcross lines in yield trial at the first cropping season in 2019

Line	Grain yield (kg ha^{-1})	Seed appearance				Palatability of head rice
		Chalky rice (%)	Seed length (mm)	Seed width (mm)	Seed length-width ratio	
SU1	9350.0	58.8	4.17	2.71	1.54	66.5
SU3	6738.6	42.3	4.25	2.61	1.63	65.0
SU8	4147.0	55.8	4.13	2.55	1.62	61.0
SU9	4692.6	56.7	4.19	2.61	1.61	66.5
SU10	4026.0	21.0	4.29	2.62	1.64	65.5
SU15	3746.6	56.7	4.32	2.66	1.62	73.0
SU19	4320.8	44.5	4.24	2.70	1.57	71.0
SU33	3667.4	55.4	4.14	2.58	1.60	-[Z]
SU39	5130.4	56.6	4.20	2.75	1.53	71.0
SU40	4483.6	48.7	4.30	2.56	1.68	74.0
SU42	5990.6	37.2	4.23	2.61	1.62	67.0
SU45	6063.2	64.9	4.20	2.60	1.62	65.0
SU46	5687.0	38.1	4.22	2.57	1.64	60.0
SU48	5999.4	30.5	4.27	2.63	1.62	75.0
SU49	8338.0	33.7	4.26	2.55	1.67	71.0
SU52	6930.0	28.8	4.34	2.58	1.68	70.0
SU58	6516.4	27.4	4.28	2.38	1.80	64.0
SU63	4105.2	31.2	4.32	2.57	1.68	74.5
SU69	5185.4	10.7	4.31	2.49	1.73	69.5
SU76	6600.0	9.0	4.31	2.61	1.65	74.0
SU83	4655.2	10.3	4.26	2.29	1.86	73.0
SU89	4567.2	18.9	4.42	2.64	1.67	74.0
SU90	5185.4	45.5	4.22	2.69	1.57	70.5
SU92	5348.2	44.2	4.38	2.73	1.60	70.5
SU94	4840.0	28.2	4.44	2.69	1.65	66.5
SU95	5889.4	26.3	4.39	2.65	1.66	67.5
SU96	7156.6	46.8	4.29	2.69	1.59	65.5
SU98	5229.4	63.6	4.27	2.69	1.59	59.0
mean	5695.0	39.0	4.27	2.61	1.64	68.5

[Z] No data.

Table S3. The agronomic characteristics, grain yield evaluation, and the blast resistance test of IR96321-315-240, DT3, and 17 BC_3F_4 backcross lines in preliminary yield trial at the second cropping season in 2019

Line or variety	Agronomic characteristic		Grain yield evaluation		Blast resistance test	
	Plant height (cm)	Panicle number (No.)	Grain yield (kg ha^{-1})	Yield index[z] (%)	The blast code	The resistance scale
IR96321-315-240	90.2	19.2	7586.2	102.7	1	R
DT3	112.0	8.8	7385.7	100.0	4	MR
SU1	109.0	10.4	6826.5	92.4	1	R
SU3	110.4	8.0	6662.9	90.2	1	R
SU9	112.8	8.4	6662.9	90.2	1	R
SU39	120.0	8.2	6882.9	93.2	1	R
SU40	109.4	8.0	6725.7	91.1	1	R
SU42	104.2	9.4	6505.7	88.1	1	R
SU48	93.4	9.8	4557.1	61.7	4	MR
SU49	104.0	9.2	6211.8	84.1	4	MR
SU52	105.2	9.6	4808.6	65.1	1	R
SU58	115.8	8.6	5908.6	80.0	1	R
SU69	107.0	10.0	6034.3	81.7	1	R
SU76	106.2	9.2	6505.7	88.1	1	R
SU83	106.0	10.6	5758.8	78.0	1	R
SU89	110.2	8.4	7668.6	103.8	1	R
SU92	112.4	11.2	7668.6	103.8	1	R
SU94	107.4	9.8	6757.1	91.5	1	R
SU96	110.4	13.0	7857.1	106.4	1	R

[Z] The yield indices are compared with that of DT3.

Table S4. The yield components of IR96321-315-240, DT3, and 11 selected BC_3F_4 backcross lines in preliminary yield trial at the second cropping season in 2019

Line or variety	Panicle length (cm)	Panicle weight (g)	Spikelets per panicle (No.)	Seed set (%)	1000-grain weight (g)
IR96321-315-240	22.40	3.48	172.90	96.76%	20.3
DT3	22.76	4.28	177.05	85.82%	25.4
SU1	22.32	4.29	164.30	86.93%	29.8
SU3	23.72	4.59	175.15	91.04%	28.1
SU9	21.83	4.25	176.15	90.19%	19.0
SU39	21.38	3.73	153.40	85.76%	26.1
SU40	23.12	3.78	168.50	88.82%	23.8
SU58	24.54	4.44	185.10	92.67%	24.3
SU69	21.18	3.49	154.95	87.99%	25.6
SU89	21.12	3.56	148.56	88.58%	24.4
SU92	23.01	4.22	163.30	93.56%	26.0
SU94	23.66	4.96	180.50	92.43%	26.3
SU96	21.84	4.46	152.15	95.57%	29.0

Table S5. The seed appearance and palatability of IR96321-315-240, DT3, and 11 selected BC_3F_4 backcross lines in preliminary yield trial at the second cropping season in 2019

Line or variety	Seed appearance					Palatability of head rice
	Chalky rice (%)	Seed length (mm)	Seed width (mm)	Seed length-width ratio	Seed thickness (mm)	
IR96321-315-240	0.4%	4.92	2.02	2.44	1.77	44.0
DT3	10.3%	4.81	2.92	1.65	2.06	83.5
SU1	20.3%	4.86	2.94	1.65	2.05	79.0
SU3	14.6%	4.98	2.88	1.73	2.04	81.5
SU9	14.0%	4.88	2.89	1.69	2.06	82.5
SU39	14.0%	4.84	2.98	1.62	2.06	86.0
SU40	3.4%	5.07	2.58	1.97	1.92	87.5
SU58	15.1%	4.74	2.58	1.84	1.97	75.0
SU69	3.9%	4.90	2.59	1.89	1.88	74.0
SU89	4.2%	4.87	2.87	1.70	2.02	80.5
SU92	6.9%	4.91	2.86	1.72	1.98	84.0
SU94	8.6%	4.81	2.90	1.66	2.04	72.5
SU96	12.5%	4.87	2.89	1.69	1.99	78.0

Table S6. Details of functional and linkage markers and their primer sequences used for foreground selection.

Markers	Primer sequence	Annealing temperature (℃)
Sub1A1 (F)	CTTCTTGCTCAACGACAACG	50.9
Sub1A1 (R)	TCGATGGGGTCTTGATCTCT	50.8
Sub1AB1(F)	CATGTTCCATAGCCATCGACT	51.6
Sub1AB1(R)	GAGCGAAGAGAGCTACCTGAA	50.7
